# Association Analysis between SNPs in IL-28B Gene and the Progress of Hepatitis B Infection in Han Chinese

**DOI:** 10.1371/journal.pone.0050787

**Published:** 2012-12-05

**Authors:** Jie Chen, Lanlan Wang, Yi Li, Bei Cai, Yang Fu, Yun Liao, Junlong Zhang

**Affiliations:** Department of Laboratory Medicine, West China Hospital, Sichuan University, Chengdu, P. R. China; The University of Texas Health Science Center, United States of America

## Abstract

**Objective:**

As a candidate gene association study, we investigated the genetic association of SNPs in IL-28B genes with different outcomes of HBV infection, including LC and HCC occurrence.

**Methods:**

Chinese Han subjects were categorized into two groups: 406 LC caused by CHB and 406 HCC caused by CHB. Genomic DNA was isolated from whole blood samples, SNPs were detected using high resolution melting curve (HRM) method. PCR amplification was carried out under the same conditions in a 96-well plate in Real-Time PCR System. Then 341 LC and 356 HCC patients caused by HBV infection were analyzed as a verification by independent sample. 393 CHB patients and 244 health subjects were included as control.

**Results:**

CHB patients who progress to LC or HCC showed a significant different frequency in rs12979860 (*p* = 0.046). Patients with HCC carried more frequently the T alleles in rs12979860 comparison to LC. Same results were found in the independent sample.

**Conclusion:**

IL-28B rs12979860 C/T polymorphism T allele appears to be more prevalent in patients with HCC than in LC. Carriage of this allele seems to enhance the risk for developing HCC. Gene polymorphism of IL-28B may confer symptomatic specificity in progress and extent of hepatitis B infection.

## Introduction

More than 2 billion people have been infected with the hepatitis B virus (HBV) worldwide, of which 350 million are chronic carriers and about 600 000 die annually of HBV-related acute or chronic liver disease[1]. Although many individuals eventually achieve a state of nonreplicative infection, the prolonged immunologic response to infection leads to the development of cirrhosis, liver failure, or hepatocellular carcinoma (HCC) in up to 40% of patients. HCC is the third most common cause of cancer-related death, and more than 80% of HCC cases can be attributable to chronic infection with HBV in hyper-endemic regions, suggesting CHB was a major risk factor for development of HCC[2].

The enormous variation in clinical outcome of HBV infection highlights the importance of identification of mechanism underlying the progression of HBV exposure to CHB for prevention against HBV-induced fatal liver disease. Although the environmental factors such as alcohol abuse, infection age, and co-infection with other hepatitis virus unveiled as risk factors of HBV-induced liver disease, genetic factors may also influence clinical progression after HBV exposure, which is indicated by familial studies[3]. In fact, multiple candidate genes have been extensively investigated in the progression to CHB, but results were inclusive. However, no comprehensive analysis has been performed to explore this genetic variant on the progression of CHB.

IL-28B, in addition to IL-28A and IL-29, belongs to the type III IFN family, also named IFN-λ, the genes of this family of cytokines cluster on human chromosome 19[4]. It was reported that IL-28B gene polymorphisms play an important role in virus clearance in hepatitis C virus (HCV) and hepatitis B virus (HBV) infection [5]. Beside its antiviral properties, IFN-λ exhibits anti-tumor activity; in fact, several experimental studies demonstrated that the activation of type III IFN induces apoptosis and possesses anti-tumor activities[6]. Whether IL-28B gene polymorphism is associated with the outcome of chronic viral hepatitis is unclear. Martin and their group reported that IL28B single-nucleotide polymorphism affects the immune response to HCV but not to HBV or HIV[7], but Fabris reported that IL-28B rs12979860 C/T polymorphism T allele is more prevalent in bad progress[8]. This contradictory result is very interesting, and Asian population study was seldom reported, one said T allele and non-CC genotypes have strong predictive effect of increasing susceptibility of chronic HBV infection and HCC[9]. The frequency of IL-28B(rs12979860C/T) CC homozygosity was significantly different in these studies, race difference and sampling error maybe the reason.

If carriage of specific alleles truly affects response to IFN, and IFN therapy changes the natural history of chronic viral hepatitis, therefore patients with favorable alleles would have mild outcome, while patients with unfavorable alleles would progress to worse outcome. Based on these premises, we evaluated IL-28B gene polymorphism allele distribution in a series of patients with LC (liver cirrhosis) and HCC caused by HBV. Because of the connection between these SNPs and HBV infection, we included CHB patients and health subjects as control to exclude the hypothesis of the SNPs and association with HBV infection.

## Materials and Methods

### 1. Patients

Chinese Han subjects were categorized into two groups: 406 LC caused by CHB and 406 HCC caused by CHB. All diagnosis of HCC and LC were defined by clinical and biological criteria and confirmed by image technologies (abdominal ultrasound examinations, computed tomography and echography). To exclude other host risk confounders involved in HCC development, all the patients selected here were free of other hepatic virus co-infection, alcohol consumption, and with no sign of autoimmune disease. Age distribution and gender composition were matched in the groups. All patients lived in the same geographical area. All patients included in this study were hepatitis B surface antigen (HBsAg) positive over a 6-month period. Patients were positive for anti-HBs without anti-HBc (serological status by hepatitis B vaccination). The protocol was approved by the ethics committee of west China Hospital, and all patients provided informed consent before enrollment. The base characteristics of the patients were shown in table 1. Then we cooperated with the department of laboratory medicine in the first affiliated hospital of Chongqing medical university(in a different province of China). They friendly provided us 341 LC and 356 HCC patients caused by HBV infection. We analyzed the genotype of these patients as a verification of independent sample. 393 CHB patients and 244 health subjects were also included as control.

**Table 1:** Demographic characteristics of LC and HCC patients caused by hepatitis B.

characteristics	LC(n = 406)	HCC(n = 406)	P
Age(yr)	47(37–64)	49(40–63)	0.583
Gender (M:F)	271/135	290/116	0.149
HBV copies (×10^3^copies/mL )	1.03(1–62)	1.02(1–10.3)	0.384
HBs-Ag positive	329/406	321/406	0.482
HBe-Ab positive	233/406	251/406	0.198
Alpha Fetoprotein(AFP)	4.27(1.65,9.23)	78.4(3.43,841.5)	0.000*
Total Protein(TP)	56.4(51.3,66.2)	63.4(55.5,67.5)	0.430
Albumin(ALB)	29.4(27.9,34.6)	32.9(31.1,39.2)	0.003*
Total Bilirubin(TBIL)	30.4(16.8,57.4)	19.2(11.9,25.4)	0.000*
Direct Bilirubin(DB)	16.2(7.4,32.9)	7.7(5.2,13.2)	0.000*
Alanine Aminotransferase(ALT)	41(23,64)	62.5(35,102)	0.027*
Aspertate Aminotransferase(AST)	55(34,89)	71(34,122)	0.235
alkaline phosphatase(ALP)	93(68,159)	108(81,172)	0.241
gamma-glutamyl transpeptidase(GGT)	40(21,113)	64(32,143)	0.024*
prothrombin time (PT)	14.2(16.3,18.2)	12.4(11.7,13.6)	0.000*
International Normalized Ratio(INR)	1.41(1.27,1.61)	1.12(1.03,1.31)	0.000*
Activated partial thromboplastin time(APTT)	39.1(33.2,45.5)	28.3(25.3,34.6)	0.000*
Fibrinogen(FIB)	1.67(1.2,2.5)	3.13(2.27,3.86)	0.000*

Note:values are shown as mean ± SD or median (range). Data were analyzed by chi-squre analyses test and Mann–Whitney U test.

* :P<0.05.

### 2. Genomic DNA extraction

Blood samples (3 mL) were collected in EDTA tubes, and genomic DNA was isolated from whole blood samples using the whole blood DNA kit (Biotake corporation). DNA was extracted from 200 µL of the whole blood according to the manufacturer’s protocol. The concentration of DNA was diluted to10 ng/µL for working solutions and the isolated DNA was stored at −20°C.

### 3. Polymerase chain reaction

The IL-28B gene polymorphism in the promoter region were assessed. Some samples were previously genotyped by sequencing as controls for the three SNPs. The polymerase chain reaction (PCR) and melting curve analyses were performed under the same conditions in a 96-well plate on the Light Cycler480 (Roche Diagnostics, Penzberg, Bavaria, Germany). The primers were designed into a small fragment surrounding the polymorphisms and avoiding the presence of other sequence variations in the primer region.

Primers for PCR amplification were:

rs12979860∶5′-ATTCCTGGACGTGGATGGGTAC-3′ (forward);

5′- AGCGCGGAGTGCAATTCA-3′(reverse);

rs8099917∶5′-TTGTCACTGTTCCTCCTTTTGTTT-3′ (forward);

5′-TGGGAGAATGCAAATGAGAGATA-3′ (reverse);

rs12980275∶5′-GCCAGTCTCAAAAGAACAAATGC-3′ (forward);

5′- CTACCCCGGCAAATATTTAGACA-3′ (reverse);

The reaction mixtures contained 1.0 µL purified genomic DNA (10 ng/µL), 0.5 µL forward primer, 0.5 µL reverse primer, 1.4 µL20×EVA-GREEN, 0.5 µL dNTP(10 mM), 0.2 µL Hot Star Taq® Plus DNA Polymerase, 2 µL 10×buffer and 1 µL50 mM MgCl2. Real-time PCR was performed under the following conditions: an initial denaturation step at 95°C for 15 min, continued with 50 cycles of 95°C for 10 s, 60°C for 15 s, and 72°C for 20 s. After the amplification phase, a melting curve analysis was performed at 95°C for 1 min, 40°C for 1 min, 65°C for 1 s, followed by slow heating at 0.01°C/s to 95°C.

### 4. High Resolution Melting curve (HRM) analysis

**Figure 1:**
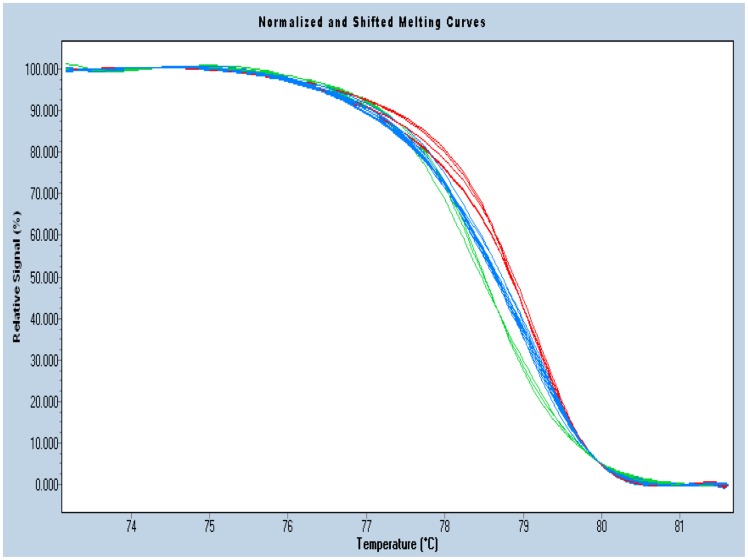
There are three groups of curves in the figure, red ones and green ones indicate homozygote, red curves on the right side indicates G or C whose Tm is high, green curves on the left side indicates A or T whose Tm is low, and the blue curves in the middle indicates heterozygote. The genotype of subset was defined according to known genotypes of controls which were determinate by gene sequencing.

Collected data were analyzed by the LightCycler 480 Gene Scanning software v1.2 (Roche Diagnostics). All the samples with amplification were monitored by real-time PCR. Software programs employed analysis below: normalization by selecting linear regions before and after the melting transition, temperature shifting by selecting threshold, then automatic grouping by calculation. The exactly same setting of the normalization was used for all experiments. The genotype of subset was defined according to known genotypes of controls which were determinate by gene sequencing. (figure 1).

### 5. Statistical analysis

Statistical analysis was performed by SPSS 16.0 (SPSS Inc., Chicago, IL). The Mann–Whitney U-test and the Kruskal–Wallis test were used for comparisons of assays results between two groups and among several groups. Data were expressed as mean±SD or median (range). The frequencies of genotype and allele were compared among patients and controls using Pearson X^2^ analysis. A P-value less than 0.05 indicated the statistical significance.

## Results

### 1. Clinical Characteristics

406 Chinese Han LC subjects caused by CHB and 406 HCC subjects caused by CHB were included in this study. There is no difference of age and gender between the two groups. There is no difference between the two groups in HBV copies and serology pattern.( Table 1).

### 2. Lab Results

Liver function tests and coagulation tests were analyzed for both groups. There is no significant difference between two groups of total protein(TP) , but albumin decreased while bilirubin increased in LC patients than in HCC ones. Alanine Aminotransferase(ALT), Aspertate Aminotransferase(AST), alkaline phosphatase(ALP) and gamma-glutamyl transpeptidase(GGT) were analyzed and all of them increased in HCC than in LC, but only ALT and GGT increased significantly(P<0.05) in HCC than in LC( Table 1).

Prothrombin time (PT), international normalized ratio (INR), activated partial thromboplastin time(APTT) and fibrinogen(FIB) were analyzed for both groups. Coagulation time increased in LC group than in HCC ones (P<0.05) ( Table 1).

### 3. Genotype

No departure from the Hardy–Weinberg distribution was observed for each genotype (p value always not significant) in control subjects and in patients with liver diseases. CHB patients who progress to LC or HCC showed a significant different frequency in rs12979860 (*p* = 0.046).Patients with HCC carried more frequently the T alleles in rs12979860 comparison to LC( Table 2). Same results were found in the independent sample.(Table 3).

**Table 2:** IL-28B SNPs in LC and HCC caused by HBV, group of CHB and health control.

polymorphism	LC(n = 406)	HCC(n = 406)	CHB(n = 393)	Health control(n = 244)	P					
					LC vsHCC	LC vshealth	LC vsCHB	HCC vshealth	HCC vsCHB	CHB vshealth
IL-28B rs12979860 C/T										
Genotypes CC	365(89.9)	348(85.7)	330(84.0)	213(87.3)	0.046*	0.305	0.013*	0.570	0.250	0.250
CT	39(9.6)	53(13.1)	60(15.3)	29(11.9)						
TT	2(0.5)	5(1.2)	3(0.8)	2(0.8)						
CT+TT	41(10.1)	58(14.3)	63(16.03)	31(12.7)						
IL-28B rs12980275 A/G										
Genotypes AA	359(88.4)	347(85.5)	330(84.0)	210(86.1)	0.211	0.378	0.068	0.833	0.556	0.474
AG	44(10.8)	57(14.0)	61(15.5)	33(13.5)						
GG	3(0.7)	2(0.5)	2(0.5)	1(0.4)						
AG+GG	47(11.6)	59(14.5)	63(16.03)	34(13.93)						
IL-28B rs 8099917 A/C										
Genotypes AA	367(90.4)	356(87.7)	352(89.6)	218(89.3)	0.217	0.666	0.697	0.524	0.402	0.929
AC	38(9.4)	48(11.8)	40(10.2)	25(10.2)						
CC	1(0.25)	2(0.5)	1(0.3)	1(0.4)						
AC+CC	39(9.61)	50(12.3)	41(10.4)	26(10.66)						

Values are shown as number (frequency), Data is analyzed by chi-squre analyses.

* refer to P<0.05.

**Table 3:** IL-28B SNPs in LC and HCC caused by HBV (verification by independent sample).

polymorphism	LC(n = 341)	HCC(n = 356)	X^2^	P
IL-28B rs12979860 C/T				
Genotypes CC	310(90.9)	306(85.96)	4.162	0.041*
CT+TT	31(9.1)	50(14.04)		
IL-28B rs12980275 A/G				
Genotypes AA	307(90.0)	304(85.4)	3.461	0.063
AG+GG	34(9.97)	52(14.6)		
IL-28B rs 8099917 A/C				
Genotypes AA	311(91.2)	312(87.6)	2.329	0.127
AC+CC	30(8.80)	44(12.4)		

Values are shown as number (frequency), Data is analyzed by chi-squre analyses.

* refer to P<0.05.

Because of the connection between these SNPs and HBV infection, we included CHB patients and health subjects as control to exclude the hypothesis of the SNPs and association with HBV infection. There is no significant difference of frequency in IL-28B (rs12979860) between HCC/LC and health control. There is significant difference between LC and CHB, but no significant difference between HCC and CHB. There is no significant difference between CHB and health control (Table 2).

### Logistic regression

Logistic regression models were used for calculating odds ratios (95% confidence interval) and corresponding P-values for each SNP site and other clinical characteristics using SPSS. We do single factor regression first, all of these variable were included: RBC,HB,PLT,WBC,TB,DB,IB, ALT,AST, TP,ALB,GLB,ALP,GGT,NH3,PT,INR,APTT,TT,FIB,AFP,HBV-DNA,HBsAg, HBsAb,HBeAg,HBeAb,HBcAb, and IL-28B genotype. Then we chose whose P<0.05 to do multi factor regression. Age (continuous value) and sex (male  =  0, female  =  1) were adjusted by inclusion in logistic analysis as covariates. Gene polymorphism in IL-28B (rs12979860) was found to be significantly associated with HCC occurrence(P = 0.038, 95%CI of OR: 1.037∼1.381), whereas no significant associations were observed between other candidate gene polymorphisms and the HCC occurrence, nor other clinical characteristics. (Table 4).

**Table 4:** Logistic regression of clinical characteristics and SNPs with HCC occurrence.

characteristics	sig	OR	OR(95%CI)
RBC	0.139	0.055	0.001∼2.574
HB	0.071	1.193	0.640∼1.364
PLT	0.110	1.016	0.911∼1.082
WBC	0.094	0.743	0.565∼1.076
TB	0.090	1.070	0.990∼1.156
IB	0.184	0.820	0.611∼1.099
INR	0.082	0.564	0.003∼1.049
TT	0.125	0.496	0.202∼1.214
FIB	0.076	0.142	0.016∼1.229
AFP	0.295	1.001	0.999∼1.003
IL-28B(rs12979860)T	0.038*	1.125	1.037∼1.381
IL-28B(rs12980275)G	0.061	0.957	0.762∼1.103
IL-28B(rs8099917)C	0.142	0.940	0.843∼1.188

Note: Logistic regression models were used for calculating odds ratios (95% confidence interval) and corresponding *P*-values for each SNP site and HCC occurrence. Age (continuous value) and sex (male  =  0, female  =  1) were adjusted by inclusion in logistic analysis as covariates. (*p<0.05).

## Discussion

Hepatocellular carcinoma (HCC) is the fifth most common cancer worldwide and the third most common cause of cancer mortality. Globally, Hepatitis B virus (HBV) is the most frequent underlying cause of HCC. In hyper-epidemic areas such as China and Africa, chronic HBV infection contributes to at least 80% of cases of HCC[10]. There is intense interest in cellular and molecular mechanisms underlying HBV-associated HCC incidence. However, due to the long duration (usually more than three decades) from HBV infection to HCC incidence and the complexity of carcinogenesis, mechanism underlies the HCC development in hepatitis B patients is poorly understood. Persistent inflammation was recognized to function as a driving force in the journey to HCC as well as in many other cancers. Longstanding liver inflammation leads to hepatic regeneration and fibrosis, which subsequently progresses to cirrhosis in some patients with chronic hepatitis B virus (HBV) infection. HBV likely causes HCC via both indirect (necro-inflammation and regeneration injury) and direct (by integration of its DNA in the host genome) pathways[11]. During the process of inflammation, cytokines play the most important role [12].

Recently, more and more attention was paid to IL-28B, it seems to play a central role in the immune response towards viral infections; in particular, type III IFN demonstrates to possess potent antiviral and immune-stimulation activities in response to poxvirus infection. IL-28B polymorphisms are linked with a better response to antiviral treatment in patients with HCV chronic hepatitis and to a higher probability to clear the virus during the natural history of chronic HCV infection[13]. We guess IL-28B may play a role in immune response of the process and progress of hepatitis. So this study wants to explore whether IL-28B polymorphisms are linked with the outcome of HBV infection and inflammation, whether IL-28B polymorphisms are linked with HCC occurrence.

We conducted in HBV-infected patients who have progressed to HCC or LC. Age and gender were matched. Liver function tests and coagulation tests were analyzed to explore the difference between stages such as LC and HCC. There is no difference between the two groups in HBV copies and serology pattern. This can exclude above inference factors. There is no significant difference between two groups of total protein(TP) , but albumin decreased while bilirubin increased in LC patients than in HCC ones. ALT and GGT increased significantly in HCC patients than in LC. This indicates that the liver injury is heavier in HCC than in LC. But jaundice degree is much light in HCC. In order to know whether the level of serum enzymes and coagulation tests are strictly associated with progress to carcinoma, we use logistic regression models to analysis which factor may be the most important one in the progress of hepatitis. Many clinical characteristics were included, such as liver function tests, serum enzymes, coagulation tests, virus DNA load, and so on. Gene polymorphism in IL-28B (rs12979860) was found to be significantly associated with HCC occurrence, whereas no significant associations were observed between other factors and HCC occurrence. The most important factors may become predictive tests for forecast unhealthy progress including carcinoma.

A novel finding concerns the demonstration of a strict association between IL-28B rs12979860 C/T polymorphism and the occurrence of HCC. The carriage of the T alleles in rs12979860 was strictly associated with the presence of HCC in our subjects in whom the presence of liver cancer was actively searched in the liver and confirmed at histology. The trends in genotype frequencies are highly significantly different between these two groups. Carriage of the T alleles in rs12979860 were found to be independent predictor of the presence of HCC in multivariate analysis. If confirmed, these data could have important clinical consequences in the selection of cirrhotic patients for whom screening for early HCC detection might need to be intensified.

This phenomenon can be explained as following: first, genotype CC was indicated to be associated with virus clearing, thus HBV may stay much longer in those patients who with the mutation allele T[14], so T alleles in rs12979860 was associated with HCC occurrence. Second, IFN therapy inhibits hepatic carcinogenesis in patients with chronic hepatitis B[15], this indicates that IL-28B with mutation type can’t function normally, they can’t inhibits carcinogenesis effectively, and resulted in malignant progress and outcome. Third, the predictive time of this test is much earlier than abdomen B ultrasonic and the results are much sensitive and specific than other tests such as liver function tests and biopsy.

In conclusion, IL-28B T alleles in rs12979860 appear to be more prevalent in patients with HCC. They are functionally important in determining disease outcome. Among patients of liver cirrhosis, especially those with HBV infection, carriage of this allele seems to enhance the risk for developing HCC. Our study is useful for HCC surveillance and has implications for novel personalized therapy strategy development aiming at HCC.
